# High-throughput screening and whole genome sequencing identifies an antimicrobially active inhibitor of *Vibrio cholerae*

**DOI:** 10.1186/1471-2180-14-49

**Published:** 2014-02-26

**Authors:** Galina Sergeev, Sambit Roy, Michael Jarek, Viktor Zapolskii, Dieter E Kaufmann, Ranjan K Nandy, Werner Tegge

**Affiliations:** 1 Department of Chemical Biology, Helmholtz Centre for Infection Research (HZI), Inhoffenstraße 7, D-38124 Braunschweig, Germany; 2 National Institute of Cholera and Enteric Diseases (NICED), P-33, CIT Road, Scheme XM Beliaghata, Kolkata 700 010, India; 3 Technical University of Clausthal, Leibnizstraße 6, D-38678 Clausthal-Zellerfeld, Germany

**Keywords:** *Vibrio cholerae*, Small molecules, Histidine kinase inhibitor, KdpD, whole genome sequencing

## Abstract

**Background:**

Pathogenic serotypes of *Vibrio cholerae* cause the life-threatening diarrheal disease cholera. The increasing development of bacterial resistances against the known antibiotics necessitates the search for new antimicrobial compounds and targets for this pathogen.

**Results:**

A high-throughput screening assay with a *Vibrio cholerae* reporter strain constitutively expressing green fluorescent protein (GFP) was developed and applied in the investigation of the growth inhibitory effect of approximately 28,300 structurally diverse natural compounds and synthetic small molecules. Several compounds with activities in the low micromolar concentration range were identified. The most active structure, designated vz0825, displayed a minimal inhibitory concentration (MIC) of 1.6 μM and a minimal bactericidal concentration (MBC) of 3.2 μM against several strains of *V. cholerae* and was specific for this pathogen. Mutants with reduced sensitivity against vz0825 were generated and whole genome sequencing of 15 pooled mutants was carried out. Comparison with the genome of the wild type strain identified the gene VC_A0531 (GenBank: AE003853.1) as the major site of single nucleotide polymorphisms in the resistant mutants. VC_A0531 is located on the small chromosome of *V. cholerae* and encodes the osmosensitive K^+^-channel sensor histidine kinase (KdpD). Nucleotide exchange of the major mutation site in the wild type strain confirmed the sensitive phenotype.

**Conclusion:**

The reporter strain MO10 pG13 was successfully used for the identification of new antibacterial compounds against *V. cholerae*. Generation of resistant mutants and whole genome sequencing was carried out to identify the histidine kinase KdpD as a novel antimicrobial target.

## Background

*Vibrio cholerae*, a Gram-negative rod-shaped bacterium belonging to the family Vibrionaceae, induces the acute diarrheal disease cholera. Cholera has pandemic properties and appears mainly in third world countries with estimated 3–5 million cases and more than 100,000 deaths per year [1]. The major pathogenic strains belong to the serogroups O1 and O139. Infections are treated by oral or intravenous rehydration therapy, which is complemented in severe cases with antibiotics to shorten the duration of the clinical symptoms and to reduce the spreading. Long-term and extensive use of antibiotics has led to resistance development. A growing problem is the emergence of multidrug resistant pathogenic *V. cholerae* strains against which therapeutic options are more and more limited [2]. Due to this development the availability of novel therapeutic options is urgently needed.

In the present study we have developed a high-throughput screening (HTS) assay that utilizes a *V. cholerae* reporter strain constitutively expressing green fluorescence protein and screened approximately 28,300 compounds from six different chemical structural groups in a growth inhibition assay. Several active molecules were identified which are active in suppressing growth of *V. cholerae in vitro. V. cholerae* mutants resistant to the most potent molecule were generated. Whole-genome sequencing and comparative analysis of the mutant to the wild type strain was carried out. The apparent target of the most active compound was identified to be the osmosensitive K^+^-channel sensor histidine kinase KdpD that apparently exerts certain essential function in this pathogen.

## Results

### HTS assay for inhibitors of *V. cholerae* viability

**Figure 1:**
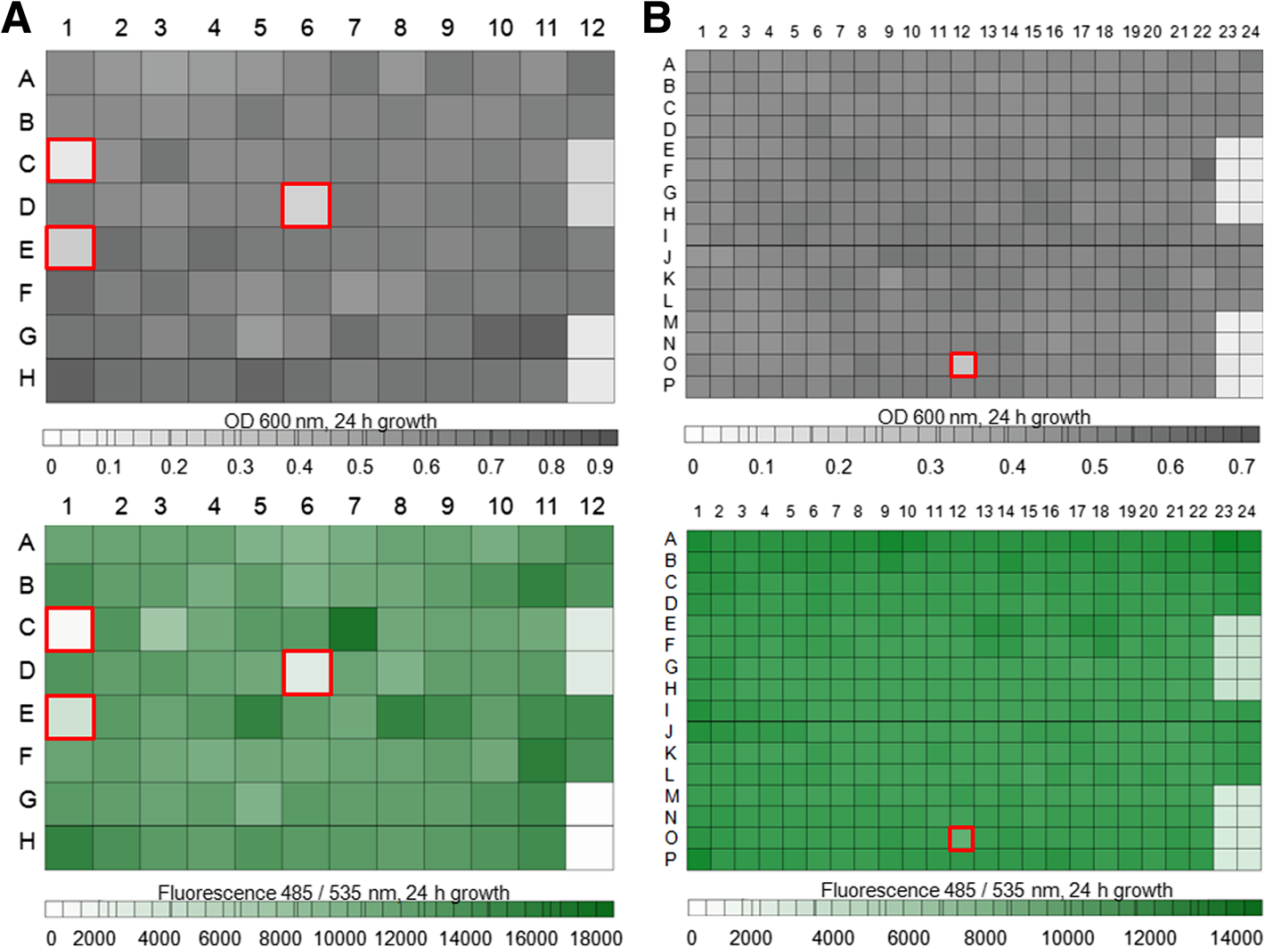
**HTS assay.** Growth of *V. cholerae* MO10 pG13 strain in 96- **(A)** and 384-well MTP **(B)** in the presence of test compounds and controls. **(A)**: 12 A-B: 1% DMSO, 12C-D: 100 μM ciprofloxacin, 12 E-F: no addition of compounds, 12 G-H: sterile medium. **(B)**: 23 A-D and 24 A-D: 1% DMSO, 23 E-H and 24 E-H: 100 μM ciprofloxacin, 23 J-M and 24 J-M: no addition of compounds, 23 M-P and 24 M-P: sterile medium. Upper panels: absorbance at 600 nm; lower panels: fluorescence (485/535 nm). Wells framed in red indicate active compounds.

Green fluorescence producing plasmid pG13 was electroporated into *V. cholerae* strain MO10 and the transformants were selected on LB agar plates containing kanamycin (Km, 30 μg/ml). Transfer of the plasmid pG13 conferred green fluorescence phenotype in *V. cholerae* O139 strain MO10. The screening assay was optimized in 96- and 384-well microtiter plates (MTP). To differentiate between active and non-active compounds and as controls for the functionality of the assay, ciprofloxacin (Cip, 100 μM) and dimethyl sulfoxide (DMSO, 1%) were included on each plate. DMSO had no growth reducing effect at concentrations up to 1%. The evaluation of the effect of compounds on the growth of strain MO10 pG13 was carried out after 24 h of incubation, with measurement of absorbance at 600 nm in combination with fluorescence determination (Figure 1). In the screening campaigns of the six different substance collections with 28,300 compounds in total, Z’-values between 0.5 and 0.9 with a mean of 0.8 were obtained, which is an indication of a reliable performance of the assay [3].

**Figure 2:**
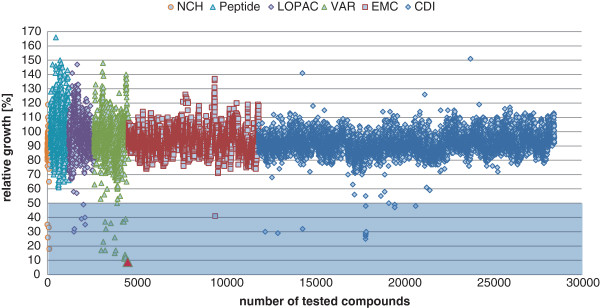
**Screening results.** Summary of the initial screening results for novel antibacterial compounds. The tested compounds came from the NCH, Peptide, LOPAC, VAR, EMC and CDI collections. The shaded area highlights the activities that were defined as initial hits. The most active compound, vz0825, stemming from the VAR collection, is highlighted in red.

The six groups of screening compounds consisted of: i) the commercially available LOPAC library (a collection of pharmaceutically active compounds); ii) and iii) the EMC (Echaz Microcollection) and CDI collections (Chemical Diversity Lab), which contain small organic molecules that were mainly generated by combinatorial synthesis; iv) the VAR collection (various sources), which is unique at the HZI and consists of small organic molecules that were synthesized by cooperating chemists; v) the NCH collection (natural compounds), which is also unique at the HZI and consists of purified secondary metabolites from myxobacteria. It included potent agents with already known antimicrobial or antiproliferative activity, e.g. epothilon, which has been developed into a therapeutic agent against breast cancer [4,5]; and finally vi) collections of linear and cyclic peptides with a length of seven or eight D- or L-amino acids were investigated [6]. The compounds were used in one defined concentration between 20 to 50 μM in the initial screening. An overview of the growth-reducing activities of the six different substance collections is shown in Figure 2 and in Table 1. The threshold for active compounds was defined at a minimum growth reduction of 50% in comparison to the DMSO control, which resulted in a suitable initial hit rate. The smallest of the six collections, the NCH collection of 154 compounds, showed the most active molecules with 32.5 hits per 1,000 substances. Several of these molecules displayed antibacterial activities that have been known before [7]. The VAR library consists of molecules with predominantly unexplored activities and contained 8.8 antibacterial compounds per 1,000 molecules. With 17 hits this collection contained the highest number of antibacterial molecules in total.

**Table 1:** Summary of the screening for growth-reducing compounds

** Substance collection (number of compounds)**	**Number of active compounds at different growth reduction rates**					**∑**	**Hit rate/1,000**
	**50-60%**	**60-70%**	**70-80%**	**80-90%**	**90-100%**		
NCH (154)	0	2	1	2	0	5	32.5
Peptide (1,045)	0	0	0	0	0	0	0
LOPAC (1,408)	2	4	0	0	0	6	4.3
VAR (1,936)	1	5	2	8	1	17	8.8
EMC (7,304)	1	0	0	0	0	1	0.1
CDI (16,608)	5	3	5	0	0	13	0.8
28,324						42	1.6

**Figure 3:**
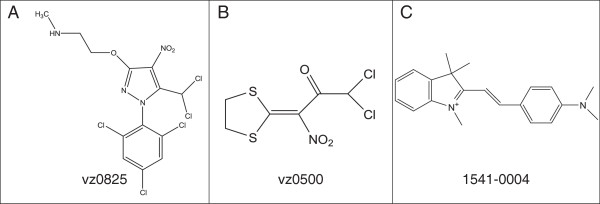
**Chemical structures.** Most active compounds of *V. cholerae* growth inhibition. Panel **A**: compound vz0825; Panel **B**: compound vz0500; Panel **C**: compound 1541-0004.

In total 42 hits were identified in the initial screening campaign. These initial hits were reevaluated in different concentrations by using *V. cholerae* strains and several other Gram-positive and Gram-negative pathogenic bacteria. After these reevaluations, the number of active compounds was reduced to three most promising agents with the designations vz0825, vz0500 and 1541–0004. The former two compounds are derived from the VAR library, the last one from the commercially available CDI library. The chemical structures are shown in Figure 3.

### MIC and MBC values of the most active substances

The two pathogenic *V. cholerae* O1 type stains N16961 and NM06-058 were used to determine the MIC and MBC values for the compounds vz0825, vz0500 and 1541–0004 (Table 2). *V. cholerae* N16961 belongs to biotype El Tor which caused the seventh pandemic [8] and was isolated in 1971. *V. cholerae* NM06-058 was isolated in 2006 in Kolkata from a cholera patient and represents the altered El Tor biotype. The active compounds inhibited growth of both strains equipotent at low micromolar concentrations with MIC values of 1.6 μM, 3.1 μM and 6.3 μM, respectively*.* In order to obtain reliable data, bactericidal activities were determined after 2, 6 and 24 hours. All three compounds killed the bacteria at low micromolar concentrations, only slightly above the respective MIC values (Table 2). Further nine *V. cholerae* strains belonging to the O1, O139 and non O1/O139 serogroups (Table 3) (three strains of each serogroup) were testes with compound vz0825, which is active against all tested strains with MIC values between 0.4 and 3.1 μM. Overall vz0825 was the most active substance.

**Table 2:** **MIC and MBC values for the most active compounds against *V. cholerae***

			**Concentration [μM]**		
***V. cholerae *strain**		**Incubation time**	**vz0825**	**vz0500**	**1541-0004**
**N16961**	MIC	24 h	**1.6**	**3.1**	**6.3**
	MBC	2 h	50	50	50
		6 h	12.5	6.3	6.3
		24 h	6.3	6.3	6.3
**NM06-058**	MIC	24 h	**1.6**	**3.1**	**6.3**
	MBC	2 h	50	50	6.3
		6 h	12.5	6.3	6.3
		24 h	1.6	6.3	6.3

**Table 3:** Strains, cells, plasmids and primers used for this study

**Strain, cell, plasmid, primer**	**Relevant description/sequence**	**Reference or source**
**Strains**		
*V. cholerae*		
MO10 pG13	O139 containing pG13	This study
N16961	Wild type, O1, El Tor, Inaba	Makassar (1971), clinical isolate [8]
NM06-058	Wild type, O1, El Tor, Ogawa	Kolkata (1996), clinical isolate
NM06-058 T283M	Contains a point mutation in gene VC_A0531 on AA position 283	This study
RKI-ZBS2-A310-3	Isolate, O1, El Tor, Inaba	RKI
RKI-ZBS2-A310-12	Isolate, O1, El Tor, Ogawa	RKI
RKI-ZBS2-A198-1	Isolate, O1, El Tor, Ogawa	RKI
RKI-ZBS2-A310-25	Isolate, O139, El Tor	RKI
RKI-ZBS2-A186-9	Isolate, O139, El Tor	RKI
RKI-ZBS2-186-10	Isolate, O139, El Tor	RKI
RKI-ZBS2-A220-1	Isolate, Non O1/O139	RKI
RKI-ZBS2-A222-1	Isolate, Non O1/O139	RKI
RKI-ZBS2-A227-1	Isolate, Non O1/O139	RKI
**Gram-negative**		
*Acinetobacter baumannii*	ATCC 30007	DSMZ
*E. coli*	ESBL, 5044257621-1	HZI
*E. coli*	ETEC	NICED
*E. coli*	S17-1	HZI
*Klebsiella pneumoniae*	50219455	HZI
*Pseudomonas aeruginosa*	90013687	HZI
*Salmonella typhimurium*		NICED
*Shigella boydii*		NICED
*Shigella flexneri*		NICED
**Gram-positive**		
*Enterococcus faecalis*	ATCC 20212	HZI
*Staphylococcus aureus*	MRSA, N315	HZI
**Cell line**		
L929	Mouse fibroblastic cell line	Derived from commercial source, DSMZ: ACC 2
**Plasmid**		
pG13	Plasmid containing the constitutive expressing G13 promoter- and *gfp*-gene sequence, ligated in pFPV27 vector, (Km^r^)	[9]
pEX18Ap	Plasmid containing Amp^r^ gene β-lactamase, the sacB gene encoding the levansucrase	HZI
**Oligonucleotide primer**		
VC_A0531_forw2	TCACGAACCAACAGGATTAAG	Used for colony PCR and sequencing of the products
VC_A0531_rev2	CGGTTAAAGTGGTAGCAGAG	Same as above
Mut_forw_1	ACATCATCTAGAGCAGCAGCAACACAAGA (XbaI)	Used for generation of the point mutation
Mut_rev_1	ATCGCGCCAAGCGGC**A**TTTTTAGATCG	Same as above
Mut_forw_2	CGATCTAAAAA**T**GCCGCTTGGCGCGAT	Same as above
Mut_rev_2	ACATCAAAGCTTAACATGCGCCACCAGAC (HindIII)	Same as above
*kdpD*_del_forw_1	ACATCATCTAGAGGAATCCATCAAAGAAA (XbaI)	Used for generation of the deletion mutation of *kdp*D
*kdpD*_del_rev_1	ACAGGATTAAGAAGCAATGAACAGTGAAATTAAGATCCTC	Same as above
*kdpD*_del_forw_2	GAGGATCTTAATTTCACTGTTCATTGCTTCTTAATCCTGT	Same as above
*kdpD*_del_rev_2	ACATCACTGCAGAACACAAGATCCAACAC (PstI)	Same as above

The antibacterial specificity of the active substances was investigated with different Gram-positive and Gram-negative pathogenic bacteria, which are able to induce serious gastrointestinal infections in humans (Table 4). Apparently, the antimicrobial activity of the three substances was limited to *V. cholerae*, only compound 1541–0004 also displayed a moderate activity against *S. aureus* with an MIC of 6.3 μM.

**Table 4:** MIC values of active compounds for different pathogenic bacteria

	**MIC [μM]**		
**Bacterial strain**	**vz0825**	**vz0500**	**1541-0004**
**Gram-negative**			
*Acinetobacter baumannii*	50	> > 100	> 100
*Escherichia coli,* ESBL	> 100	> > 100	> 100
*Escherichia coli,* ETEC	> > 50	> > 50	> 50
*Klebsiella pneumoniae*	100	> 100	100
*Pseudomonas aeruginosa*	> > 100	> > 100	> > 100
*Salmonella typhimurium*	> > 50	> > 50	> > 50
*Shigella boydii*	> > 50	> > 50	> 50
*Shigella flexneri*	> > 50	> > 50	> 50
**Gram-positive**			
*Enterococcus faecalis*	50	> > 100	> 100
*Staphylococcus aureus,* MRSA	50	100	6.3

### Cytotoxicity determination via MTT-assay

*In vitro* cytotoxicity determination by MTT test with mammalian cells is one of the standard procedures for the evaluation of new active agents [10]. The well established assay was carried out with the permanent mouse fibroblast cell line L929 according to a published procedure [11] with some modifications [12]. In the assay cell viability is determined by the reduction of the yellow MTT (3-(4,5-dimethylthiazol-2-yl)-2,5-diphenyltetrazoliumbromid) to the violet formazan by the action of ER- and mitochondrial enzymes. Concentrations of the active compounds vz0825, vz0500 and 1541–0004 from 0.003 to 370 μM were used and effects on the fibroblasts were analyzed after 24 hours and 5 days of incubation. The IC_50_ values are shown in Table 5. The two most active compounds vz0825 and vz0500 showed cytotoxic (inhibition after 24 hours of incubation) and anti-proliferative (inhibition after 5 days of incubation) IC_50_ values at low micromolar concentrations. Compound 1541–0004 is less cytotoxic, but has also a strong antiproliferative activity.

**Table 5:** Cytotoxic (24 h) and antiproliferative (5 d) activity of the most active compounds according to MTT test with L929 cells

**Compound**	**IC**_**50 **_**[μM]**	
	**24 h**	**5 d**
vz0825	14	6
vz0500	3	1
1541-0004	170	14

### Generation of resistant mutants against vz0825

Mutants against vz0825 were generated by selection of variants of the wild type strain NM06-058 that are able to grow on agar plates containing 8 μM vz0825. After one round of selection, 15 resistant mutants were picked and analyzed individually. They displayed 4–16 fold reduced sensitivities (MIC 6.3 - 25 μM) against vz0825 compared to the wild type strain. In order to obtain an indication if vz0825 has a mode of action that is different from standard antimicrobials, eight established antibiotics against the major different antibacterial targets were tested with the resistant mutants. The addressed targets and their inhibitors were i) cell wall synthesis (ampicillin), ii) protein biosynthesis (tetracycline), iii) DNA-replication (ciprofloxacin), iv) DNA-dependent RNA polymerase (rifampicin), v) translation (chloramphenicol, erythromycin) and vi) synthesis of folic-acid (trimethoprim/sulfamethoxazol).

The *V. cholerae* wild type strain NM06-058 and resistant mutants did not show differences in their MIC values against all tested antibiotics (data not shown), suggesting that vz0825 has a mode of action that is different from the classical antibiotics.

### Target identification

This result initiated a further investigation of the mode of action of vz0825 by the comparative genome sequence analysis approach. The method makes use of whole genome sequence analysis of resistant mutants that were generated against an active compound and the comparison of the genome of the wild type and the mutant strain [13]. The genomes of the 15 resistant *V. cholerae* mutants were isolated, pooled and analyzed via paired-end sequencing. In parallel, the genome of the wild type strain from which the resistant mutants have been generated was also sequenced by the same method. The alignment and annotation of both probes was based on the published genome of *V. cholerae* strain N16961 (chromosome 1: AE003852, chromosome 2: AE003853 in NCBI) [14]. As shown in Table 6, approximately 98% and 94% of the fragments from the mutant-pool and the wild type, respectively, could be aligned. The alignment was carried out via the application of CLC Genomics Workbench V. 4.7.2 software. The algorithm to search for crucial distinctions were parameters like single nucleotide polymorphism (SNP) and deletion and insertion polymorphism (DIP), where one nucleotide was affected with a minimal mutation frequency of 30%.

**Table 6:** Summarized statistics of genome sequencing

	**Number of fragments**	**Average length [bp]**	**Total base number**
**wt genome**			
Fragments	11,260,864	76	855,825,664
Identified	10,574,557 (93.9%)	76	803,666,332
Non-identified	686,307 (6.1%)	76	52,159,332
**Genome-pool**			
Fragments	35,196,596	72.36	2,546,713,435
Identified	34,210,563 (97.8%)	72.43	2,477,950,102
Non-identified	986,033 (2.8%)	69.74	68,763,333
**Reference**	2	2,016,732	4,033,460

Reference genome came from *V. cholerae* strain N16961 [14].

Under those conditions, the comparison of the wild type and the pooled sequences from the mutants showed only one significant mutation, this was located at position 848 in gene VC_A0531 and was present in about 30% (precisely 29.1%) of the sequenced fragments. These mutants have the nucleobase thymine instead of cytosine on position 848. The point mutation of this nucleobase leads to an exchange of threonine to methionine on position 283 (T283M) of the expressed protein.

The gene VC_A0531 (GenBank: AE003853.1) is located on the small chromosome of *V. cholerae* and encodes a sensor histidine kinase, which is the homologous to KdpD of *E. coli* and is responsible for osmotic potassium regulation in the bacterial cell [15]. In addition to the whole genome pool sequencing, the gene VC_A0531 (*kdpD*) of the 15 mutants was analyzed individually by PCR amplification. 4 of the 15 mutants, corresponding to 26.7%, had the same mutation on reference position 848 of the gene *kdpD* that was identified in the whole genome pool sequencing. Another four of the mutants showed point mutations at other positions of the *kdpD* gene (Table 7).

**Table 7:** **Modifications detected in gene VC_A0531 (*kdpD*) by PCR analysis of 15 resistant mutants (AA, amino acid)**

	**Nucleotide pos.**	**Ref. allel**	**Mut. allel**	**Number of mutants**	**Codon old**	**Codon new**	**AA pos.**	**AA old**	**AA new**
1	218	T	C	1	CUA	CCA	73	Leu	Pro
**2**	**848**	**C**	**T**	**4**	**ACG**	**AUG**	**283**	**Thr**	**Met**
3	1,022	C	A	1	CCU	CAU	341	Pro	His
4	1,177	G	A	1	GAA	AAA	393	Glu	Lys
5	1,178	A	G	1	GAA	GGA	393	Glu	Gly

In bold the major statistically significant mutation is highlighted.

### Sensitivity of strain NM06-058 T283M against vz0825

**Figure 4:**
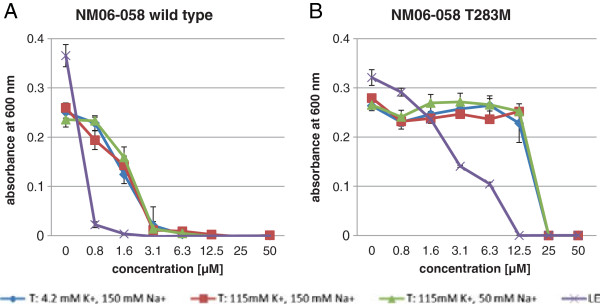
**Growth determination.** Growth of *V. cholerae* wild type strain NM06-058 **(A)** and the T283M exchange mutant **(B)** in the presence of vz0825 in media with different K^+^ and Na^+^ concentrations.

A strain containing the point mutation T283M in the *kdpD* gene was generated by site-directed mutagenesis. Successful cloning was verified by a PCR amplification of the affected gene and the sequencing of the fragment. The mutant was selected on LB-agar plates containing vz0825 at 16 μM concentration, which is 10-times higher than the MIC of the wild type strain. A growth analysis with this strain was carried out in vz0825 supplemented LB-medium and in T-medium with different potassium and sodium ion concentrations (Figure 4). Overall, growth of the T283M mutant was much less effected by vz0825 in comparison to the wild type strain. Sensitivity of the T283M mutant against compounds vz0500 and 1541–0004 did not differ from the wild type strain NM06-058 (data not shown).

### Attempts to construct a *kdpD* knockout mutant

For a further elucidation of the effect of vz0825, the construction of a *V. cholerae kdpD* knockout mutant was attempted. If KdpD is a major target of compound vz0825, the *V. cholerae kdpD* knockout mutant should be insensitive to the compound, unless the protein itself and its function are essential for the viability of the bacteria. The cloning procedure delivered the expected plasmid construct according to sequencing. The plasmid was successfully transformed into the *E. coli* strain S17-1, according to the acquirement of ampicillin resistance, which is located on the plasmid pEX18Ap and also according to PCR amplification of the construct. The conjugation of the transformed *E. coli* with *V. cholerae* and the following selection on LB agar plates supplemented with carbenicillin (Carb) and Km did not lead to clones with a deleted VC_A0531 gene, even after several modifications of the protocol. A possible explanation is that the gene product KdpD is indeed essential for *V. cholerae*, in agreement with KdpD being a prime target of vz0825.

## Discussion

A HTS assay for small molecule inhibitors of *V. cholerae* was developed and validated using a viability phenotype of *V. cholerae* that constitutively expresses green fluorescence. The assay is reliable, reproducible and simple to perform. During the development of the reporter strain, two reference strains of O1 serogroup belonging to biotypes O395 (classical) and N16961 (El Tor) were included along with the O139 strain MO10. The green fluorescence producing plasmid pG13 was electroporated into the three strains. During initial standardization experiments it was observed that the strain MO10 pG13 produced much greater level of green fluorescence as compared to other two strains (data not shown). For this reason strain MO10 pG13 was used in the screening experiments.

A data bank search in SciFinder for the most active compounds vz0825 and vz0500 did not reveal pre-described antibacterial activities of the compounds with structural similarities above 70%. Compound 1541–0004, stemming from the commercial CDI collection, belongs to the group of styryl dyes, which have already in 1966 been shown to possess antimicrobial effects against the plant pathogen *Xanthomonas oryza*[16]. The IC_50_ value for acute cytotoxicity of compound vz0825 in an MTT test was approximately 17-times higher than its MIC value. For vz0500, both IC_50_ and MIC values were about equal. For compound 1541–0004 the IC_50_ value for cytotoxicity was approximately 27-times higher than the MIC value. Although the identified compounds exhibited antimicrobial activities at low concentrations, the toxicities render them unsuitable for direct clinical application. Thus, the compounds may serve as pharmaceutical leads and modifications via the methods of medicinal chemistry may lead to better properties.

The elucidation of the mode of action of new antimicrobials can be a tedious and time consuming effort and can require the application of a variety of biochemical and molecular methods [17,18]. Due to the advances in genome sequencing instrumentation and methodology, an innovative new option has become available recently. It employs genomic sequence comparison of resistant mutants with wild type strains and has been successfully applied for target identification in a limited number of previous investigations by other researchers [13]. As we have used NM06-058 for the evaluation of the active compounds, we have used the same strain to create resistant mutants against vz0825. The *V. cholerae* strain NM06-058 was isolated from hospitalized diarrhea cases during 2006 at Kolkata, India. This strain along with other *V. cholerae* strains isolated during 2006 was studied for the expression of cholera toxin (CT) and it was identified that NM06-058 is capable of producing a higher amount of CT *in vitro* compared to other strains and to reference *V. cholerae* O1 El Tor strain N16961. Based on the high virulence expression, this strain was selected for our investigations. Clinical *V. cholerae* O1 strains isolated at Kolkata during and after 1995 belonged to altered El Tor biotypes [19]. Thus it can be considered that strain NM06-058 represents the altered *V. cholerae* El Tor biotype, which is still the prevailing type among cholera cases.

**Figure 5:**
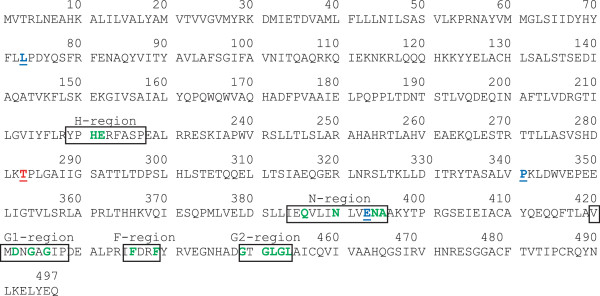
**Sequence of KdpD from *V. cholerae*.** Amino acids labeled in green in the regions H, N, G1, F, G2 are conserved in different species [20]. Labeled in red is threonine 283 which is exchanged by methionine in the dominant mutations of the resistant strains. Amino acids labeled in blue indicate the positions that are modified in four additional mutants (L73P, P341H, E393K and E393G).

The generation of mutants that were resistant against vz0825 was straightforward in this study by plating the wild type strain on agar plates containing the active compound at 5-times the MIC value of the wild type. The successful generation of resistant mutants with only one passage indicates a single essential molecular target of vz0825. The aligned sequences of the wild type genome and the mutant genome pool were compared with each other. For the identification of significant mutations the minimal frequency in the mutant genome pool was defined at 30%. A lower frequency would deliver too many non-relevant mutations. In the genome pool of the 15 resistant mutants only the gene with the code number VC_A0531, which corresponds to the homologue *kdpD* in *E.coli*, showed a significant mutation under the chosen parameters with frequency of 29.1%. The sequencing of the 15 resistant mutants showed, that 4 of them (26.7%) possess this particular modification. The mutated nucleobase is the second base of the corresponding codon and causes an exchange of the amino acid threonin by methionine in the expressed protein. Another four mutants also possess point mutations at other positions of the gene (shown in Figure 5). All of those mutations lead to an exchange of one particular amino acid in the expressed protein, two of them which are located in the N-region (position 1,177 and 1,178) lead to the exchange of glutamic acid 393 to lysine or glycin, respectively (Table 7 and Figure 5). Thus, 8 of 15 mutants possess a mutation in the *kdpD* gene.

A comparison of known protein domains in the database Pfam Protein Families [21] resulted in the localization of the affected amino acid in the dimerization/phosphor acceptor domain. Histidine kinase dimers are formed by parallel association of two domains creating 4-helix bundles; usually these domains contain a conserved histidine residue and are activated via trans-autophosphorylation by the catalytic domain [22]. They subsequently transfer the phosphoryl group to the aspartic acid acceptor residue of a response regulator protein. Based on the comparison of conserved regions in a number of bacterial histidine kinases [20], the localization could be specified more precisely between the H–region and the N-region (Figure 5). The H-region is the most variable sequence of histidine kinases in bacteria and contains the histidine that is phosphorylated in the signal transduction process. The N-region shuttles the gamma-phosphate from ATP to the histidine residue. The mutated amino acid is localized between the conserved H- and N-region (Figure 5) and thus in a part of the protein that shows high interspecies variation [23], which could explain the specificity of vz0825 against *V. cholerae*.

In the two-component system of signal transduction, the histidine kinase transfers the signal to a response regulator. The *V. cholerae* protein VC_A0531 is the homolog of KdpD in *E. coli,* the response regulator of which is KdpE [24]. The signal transduction system KdpABC, regulated by KdpD and KdpE, is part of the osmoregulation machinery in bacteria [15]. Compound vz0825 may exert its mode of action by binding to the histidine kinase KdpD and thereby inhibiting signal transduction. This would lead to a deficient uptake of potassium. If this mechanism leads to the observed reduction of bacterial viability remains to be elucidated.

Due to a lack of specific information about the potassium regulation in *V. cholerae*, we compared our findings with results that have been obtained with *E. coli. E. coli* possesses in addition to the KdpABC potassium regulatory system several further K^+^ dependent signal transduction systems. The K^+^ regulatory systems Trk and Kup are active at physiological K^+^ concentrations [15]. The expression of KdpD and consequently of the KdpABC system in *E. coli* is induced at low potassium concentrations (<60 mM) [25]. In *E. coli* KdpD is not essential at a potassium concentration >115 mM, as mutants with truncated forms of KdpD are viable under these conditions, but in media with <15 mM K^+^ those strains do not grow [25]. *V. cholerae* also possesses these three potassium regulatory systems for the adaptation to changing osmotic conditions [26,27].

The *V. cholerae* mutant strain T283M grows well in media with high and low K^+^ and Na^+^ concentrations in absence of vz0825 as shown in Figure 4. Even at 4 mM K^+^ growth is not diminished. This figure also shows the difference between the tolerance of the wild type and the T283M strain against vz0825. Our findings that T283M grows well in K^+^ reduced medium indicates that the inhibition of KdpD may have profound influence on some other, hitherto undefined, regulatory function of this protein in *V. cholerae*. The influence of vz0825 on KdpD may appear in different ways, e.g. reducing the binding of ATP to the histidine kinase, inhibiting the transfer of gamma-phosphate to the histidine residue, or to the asparagine residue of the response regulator. Like other histidine kinases KdpD also has phosphatase activity [28], which may be disturbed by vz0825. The mutated amino acid on position 283 is located between the H-region and N-region. Mutations that alter this motif, which is termed the X-region, have been shown to alter the conformation of the histidine kinase EnvZ and significantly reduce its phosphatase activity [29]. EnvZ is a membrane receptor kinase-phosphatase, which modulates porin expression in *E. coli* in response to medium osmolarity. It shares its basic scheme of signal transduction with many other sensor-kinases [29].

If KdpD is the major target of compound vz0825, the deletion construct Δ*kdpD* should be insensitive to the substance in media with physiological K^+^ concentration – provided that it is still viable. The construction of the required plasmid for the generation of this construct, its transformation into *E. coli* S17-1 and the conjugation from *E. coli* into *V. cholerae* were successful in this study, but several attempts to induce the homolog recombination within *V. cholerae* NM06-058 failed. None of the analyzed clones showed a loss of the *kdpD* gene. The apparent growth reducing effect of vz0825 and its targeting of KdpD in *V. cholerae* suggests a more important role of KdpD in *V. cholerae* than in *E. coli*. Further experiments are required in order to corroborate the effect of vz0825 on KdpD, like functional assays with the expressed protein, in which the kinase- and phosphatase activities of the wild type and mutated forms in the presence of vz0825 are compared. It would also be desirable to carry out expression profiling of the transcriptome of vz0825 sensitive and resistant *V. cholerae* strains. This procedure could help to determine how relevant the expression of *kdpD* in *V. cholerae* is and whether the expression of other genes is reduced or induced in the resistant strains.

## Conclusions

In a high-troughput screening assay with 28,300 compounds the synthetic small molecule vz0825 was identified as the most active antibacterial substance against *V. cholerae* with an MIC of 1.6 μM and an MBC of 3.2 μM. Whole genome sequencing was carried out with resistant mutants and the two-component histidine kinase KdpD was identified as the prime target of the substance. Further investigations should address the inhibitory mechanism in more detail and corroborate on the possibility of an essential function of KdpD in *V. cholerae*. Histidine kinase inhibitors are in principal promising antimicrobial drug candidates [30] and compounds like vz0825 may lead to new treatment options.

## Methods

### Strains, media and plasmids

The strains used in this study are listed in Table 3. Reporter strain MO10 pG13 was generated from the pathogenic wild type strain MO10, serogroup O139, which was electroporated with the plasmid construct pG13 containing a kanamycin resistance gene (Km^r^) and was selected on a plate containing 30 μg/ml Km. *V. cholerae* strains were grown in LB medium (pH 7.0) at 37°C. LB medium containing Km (30 μg/ml) was used for HTS and Cip (100 μM) was used for positive control. To determine the MIC and MBC values, Mueller-Hinton (MH) broth (pH 7.4) was used as growth medium. Susceptibility to ampicillin (Amp), tetracycline (Tet), Cip, rifampicin, chloramphenicol, erythromycin, sulfamethoxazole, and trimethoprim/sulfamethoxazole (SXT) was determined in 96-well MTP containing MH medium supplemented with varied amounts (1 to 1,024 μg/ml) of each antibiotic separately and varied amounts of SXT (0.13/2.38 to 8/152 μg/ml). Supplemented LB medium with Amp (50 μg/ml), Km (30 μg/ml) and Carb (100 μg/ml) was used during the procedures of site-directed mutagenesis and in T medium pH 7.4. T medium was prepared by adding 17 g tryptone, 3 g neutralized soy peptone, 10 g glucose, 50 mM MOPS, 100 mM NaCl, 2 mM KCl and 2 mM CaCl2 in 1 l of water. For homolog recombination NaCl-free (for increased sucrose sensitivity [31]) LB medium or T medium with 10% sucrose (for induction of pEX18Ap plasmid excision, carrying the *sacB* gene) was used. Cultivation of the mouse fibroblas cell line L292 was carried out in DMEM with 10% FBS (Lonza).

### Substance collections

Three commercially available substance collections were used in the screening campaigns: i) the LOPAC collection of pharmacologically active compounds with 1,408 entities (Sigma-Aldrich); ii) the *Echaz Microcollection* with 7,304 compounds (EMC Microcollections GmbH, Tübingen, Germany); and iii) the CDI collection with approximately 17,000 compounds (Chemical Diversity Lab, Inc., San Diego, USA), this commercially available collection has been assembled by members of the ChemBioNet consortium [32]. Three additional libraries that were used are unique at the HZI: iv) the NCH collection consisting of 154 secondary metabolites from myxobacteria [33]; v) the library *Various Sources* (VAR) contained at the time of this study 1,936 synthetic organic molecules that were provided by various collaborators; and vi) the *Peptide library* contained 1,045 short linear or cyclic peptide sequences synthesized at the HZI [6]. All test compounds were utilized as stock solutions in DMSO.

### Growth assay

50 μl or 25 μl of LB-Km medium were inoculated in clear flat-bottom 96-well or 384-well MTP, respectively. Test compounds were added from DMSO stocks in amounts that resulted in assay concentrations between 20 and 50 μM. 50 μl or 25 μl of bacterial culture in LB-Km medium with an absorbance of 0.2 at 600 nm (OD_600_) (Ultraspec 2100 Pro photometer, Pharmacia, GE Healthcare, Chalfont St Giles, UK) were added to the 96-well or 384-well MTP, respectively. The seeding of bacteria and addition of the compounds was carried out with the pipetting system Evolution P3 (PerkinElmer, Waltham, USA). Stationary incubation of the plates for 24 h at 37°C under moist conditions was carried out, followed by determination of absorbance at 600 nm and fluorescence at 485/535 nm (Fusion Universal Microplate Analyzer, PerkinElmer, Waltham, USA). As negative and positive controls DMSO (1%) and Cip (100 μM) were used, respectively. During the initial screening, approximately 28,300 compounds were investigated with single determinations. Compounds that reduced bacterial growth by at least 50% were retested in a second campaign and the most active substances were reevaluated at different concentrations between 0.1 and 100 μM.

### MIC and MBC values determination

The determination of MIC and MBC values was carried out with *V. cholerae* wild type strains and several Gram-negative and Gram-positive bacteria (Table 3) following standardized protocol [34] in broth dilution assays. Starting inocula of 2-8×10^5^ colony forming units/ml (CFU/ml) in MH medium at 37°C were used and serial dilutions were carried out in 96-well MTP in duplicate. At 2, 6 and 24 h of incubation, 10 μl of the cultures were plated on LB agar plates. After an incubation of the plates for 24 h at 37°C, CFU/ml were determined and used for the determination of MBC, which is defined as minimum concentration of the substance required for 99.9% reduction of CFU after an incubation period of 6 h. The 2 h and 24 h measurements were used for additional correlation. MIC values were determined after 24 h of incubation.

### Cytotoxicity assay

The mammalian cell line L929 was utilized to investigate the cytotoxicity of the active compounds in a MTT assay according to a modified protocol of Mosmann [11,12]. Following 24 h of incubation, acute toxicity was determined based on the extent of cell viability and after incubation for 5 d mainly the inhibition of cell proliferation and subacute toxicity were measured (absorption at 595 nm) (Wallac Victor 1420 Multilabel counter, PerkinElmer, Waltham, USA). IC_50_ is the concentration that reduces the viability of the cells by 50%.

### Generation of resistant mutants against vz0825

The protocol for the generation of resistant mutants was the same as used in the publication of Bielecki et al. [13]. *V. cholerae* strain NM06-058 was plated at a cell number of 1 × 10^9^ CFU on LB agar plates containing 8 μM vz0825 (5-times the MIC value). After incubation for 24 h at 37°C, micro-colonies were visible. 15 colonies were picked and preserved as mutants against vz0825.

### Isolation of genomic DNA and sequencing of genome-pool

Isolation of the genomic DNA was performed according to the protocol of the DNeasy Blood and Tissue Kit (Qiagen). Briefly, the 15 resistant mutants were inoculated individually in 5 ml LB medium and incubated for 6 h at 37°C with shaking at 180 rpm. In parallel, the wild type strain was cultivated under identical conditions. Based on the OD_600_ measurements of the cultures, the 15 mutants were pooled in equal amounts. After adjusting the cell number at 2 × 10^9^ CFU the pooled mutants and the wild type strain were collected by centrifugation. The cell pellets were lysed by addition of ATL buffer and proteinase K for 1 h at 56°C. RNA was removed by addition of 4 μl RNase A (100 mg/ml) and incubation for 2 min at RT. 200 μl AL buffer and afterwards 200 μl of ethanol were added with mixing. The mixture was transferred to DNeasy Mini spin columns and centrifuged at ≥ 6.000 × *g* for 1 min. Washing was carried out with 500 μl AW1 buffer followed by centrifugation for 1 min. A second washing step was carried out with 500 μl AW2 buffer. The tubes were centrifuged for 3 min at 20,000 × *g* and the genomic DNA was eluted from the membranes with 200 μl AE buffer.

Whole genome sequencing, alignment and annotation were carried out in the sequencing facility of the HZI (head Dr. Robert Geffers). Libraries of DNA fragments with an average length of 300 bp were prepared according the manufacturer’s instructions “Preparing Samples for Sequencing Genomic DNA” (Illumina). Sequencing was carried out with the Illumina Cluster Station and the Genome Analyzer IIx. The resulting data was transformed into FastQ-format. Sequencing of the DNA library resulted in a total base count of 855,825,664 and 2,546,713,435 for wild type and resistant mutants genome pool, respectively. This corresponds to a calculated average coverage of 214 for the wild type and for each resistant mutant to a coverage of 42. The published complete genome has a total base number of 4,033,460 (Table 6, [14]).

The sequencing procedure resulted in 11,260,862 and 35,196,596 reads for wild type and resistant mutants genome pools, respectively, which were mapped to the reference genome of the annotated *V. cholerae* strain N16961 [14] by the application of the Read Mapper Tool and the Probabilistic Variant Caller as part of CLC Genomics Workbench V. 4.7.2 software. The Read Mapper Tool maps reads and calculates average coverage at single nucleotide resolution. The Probabilistic Variant Caller identifies variants by using a probabilistic model built from read mapping data. Based on a combination of a Bayesian model and a Maximum Likelihood approach the algorithm calculates prior and error probabilities for the Bayesian model. By using the Probabilistic Variant Caller software and defining various parameters, such as sequence frequency, size of mutated areas and mutation abundance, lists of SNPs and DIPs were created. A frequency of more than 30 reads was required for all fragments. The maximum number of allel-variations was restricted to two, and the threshold of the frequency of the allel-variations was set at a minimum of 30%. These lists were compared for the wild type strain and the pooled resistant mutants, and SNPs that are unique for the mutants were identified.

### Colony PCR and sequencing

The 15 resistant mutants were analyzed individually to determine whether they carry the point mutation on position 848 of the *kdpD* gene. Individual colonies were heated in 36.5 μl of water for 5 min at 95°C. 1 μl of dNTPs (stock solution 10 mM), 2.5 μl of primers VC_A0531_forw2 and VC_A0531_rev2 (stock solution 100 pmol/μl), 5 μl 10× PCR buffer and 2.5 μl RED Taq polymerase (1 U/μl) were added. After the PCR procedure, the products had the expected size of 915 bp. They were purified and sequenced in the sequencing facility of the HZI using the above primers.

### Construction of the point-mutant KdpD T283M in strain NM06-058

The gene VC_A0531 has a size of 1,494 base pairs (coding for 497 amino acids plus stop codon). The base cytosine, which was changed to tyrosine in the predominant resistant mutants, is located on position 848. Site-directed mutagenesis was used for the incorporation of this modification into the wild type strain NM06-058. Two overlapping amplicons with a size of 525 and 616 bp were generated from the gene of the wild type strain NM06-058. Fragment one was amplified using the primer pair (i) Mut_forw_1/Mut_rev_1, and the second fragment was amplified with primer pair (ii) Mut_forw_2/Mut_rev_2. The primers Mut_rev_1 and Mut_forw_2 carried the point-mutation (Table 3, bold nucleobases). Primers Mut_forw_1 and Mut_rev_2 contained specific recognition nucleotide sequences for the restriction enzymes *Xba*I and *Hind*III. Both amplicons were mixed at equimolar ratio and a re-PCR was performed with the primers Mut_forw_1 and Mut_rev_2 to generate an amplicon with a size of 1,114 bp. This amplicon and the plasmid pEX18Ap were restricted with *Xba*I and *Hind*III. Insert and plasmid were ligated and transformed into chemically competent *E. coli* strain S17-1. Amp (100 μg/ml) was incorporated into the agar of the plate for selection of pEX18Ap containing transformants. PCR based analysis of the transformants followed by nucleotide sequencing analysis confirmed the proper insert into the vector, which was subsequently used for the conjugation assay.

Conjugation was carried out on LB agar plates overnight with a bacterial proportion of 4:1 of *E. coli* containing conjugative plasmid (donor) and *V. cholerae* as recipient strain. Bacterial cultures (mixed *E. coli* and *V. cholerae*) were plated on LB agar plate containing Carb (100 μg/ml) and Km (30 μg/ml) for selection of *V. cholerae* transconjugants carrying the plasmid. The removal of vector backbone from *V. cholerae* genome was achieved by favoring the homologous recombination and use of lethal *sacB* gene while passaging the transconjugants in sodium chloride free LB medium supplemented with 10% sucrose.

### Attempts for construction of a *kdpD* knockout mutant using *V. cholerae* strain NM06-058

The gene VC_A0531 encodes for the histidine kinase KdpD in *V. cholerae* and is flanked by the genes VC_A0530 encoding pyruvate-flavoredoxin oxidoreductase and VC_A0532 encoding response regulator KdpE homologue of *E. coli*. To generate a VC_A0531 deletion mutant, two fragments were amplified from the small chromosome of the wild type strain NM06-058 using two primer pairs (i) *kdpD*_del_forw_1 / *kdpD*_del_rev_1 and (ii) *kdpD*_del_forw_2 / *kdpD*_del_rev_2. Using the first primer pair an approximately 600 pb fragment of gene VC_A0530 was amplified with a 24 bp homolog overhang to the start region of the VC_A0532 at the C-terminus. The second primer pair was used to amplify an approximately 400 bp fragment of the gene VC_A0532 with a 16 bp overhang homolog to the end region of the VC_A0530 at the N-terminus. Both amplicons were mixed together at equimolar ratio and a re-PCR was carried out with a combination of primers *kdpD*_del_forw_1 and *kdpD*_del_rev_2 to generate an amplicon with a size of approximately 1,000 bp. The restriction of vector pEX18Ap and the insert was carried out with *Xba*I and *Pst*I. After ligation and transformation into *E. coli* S17-1, a conjugation into the wild type *V. cholerae* strain NM06-058 was mediated according to the protocol described above. The cloning strategy was successful until transconjugation according selection on Carb / Km agar plates and sequencing, but homolog recombination attempts with *V. cholerae* strain NM06-058 did not yield viable strains with deleted *kdpD* gene.

## Abbreviations

AA: Amino acid(s); Amp: Ampicillin; Carb: Carbenicillin; Cip: Ciprofloxacin; CFU: Colony-forming unit; CT: Cholera toxin; DIP: Deletion and insertion polymorphism; DMSO: Dimethyl sulfoxide; ER: Endoplasmic reticulum; GFP: Green Fluorescent Protein; HTS: High-throughput screening; Km: Kanamycin; MBC: Minimal bactericidal concentration; MIC: Minimal inhibitory concentration; MTP: Microtiter plate; RKI: Robert Koch Institute; SNP: Single nucleotide polymorphism; SXT: Trimethoprim/sulfamethoxazole; Tet: Tetracycline.

## Competing interests

The authors declare that they have no competing interests.

## Authors’ contributions

GS performed experiments, including assay development, screening, hit evaluation and the first target analysis using genome sequencing of resistant mutants. MJ is member of the sequencing facility at the HZI and carried out and interpreted the genome sequencing. SR developed the reporter strain MO10 pG13 which was used for the screening. Compounds showing activity against *V. cholerae* were conceived and synthesized by DT and VAZ. RKN and WT conceived the study, participated in its design and coordination and helped to draft or revise the manuscript. All authors read and approved the final manuscript.
